# Assessment of electronic patient education materials for adolescent bariatric surgery candidates: An environment scan

**DOI:** 10.1016/j.pecinn.2023.100143

**Published:** 2023-02-24

**Authors:** Yolanda N. Wang, Alexandra J. Heidl, Patricia M. Angeles, Biagina-Carla Farnesi, Angela S. Alberga, Tamara R. Cohen

**Affiliations:** a Faculty of Land and Food Systems, Food Nutrition and Health, The University of British Columbia, 2205 East Mall, Vancouver, BC, Canada; b School of Human Nutrition, McGill University, 21111 Lakeshore Road, Ste-Anne-de-Bellevue, QC, Canada; c Center of Excellence in Adolescent Severe Obesity, Montreal Children's Hospital, McGill University Health Center, 1001 Boulevard Décarie, Montréal, QC, Canada; d Department of Health, Kinesiology, and Applied Physiology, Concordia University, 1455 Boulevard de Maisonneuve, Montréal, QC, Canada; e Department of Pediatrics, Faculty of Medicine, McGill University, 3605 Rue de la Montagne, Montreal, QC, Canada; f BC Children's Hospital Research Institute, Healthy Starts, 938 West 28^th^ Avenue, Vancouver, BC, Canada

**Keywords:** Obesity, Severe obesity, Bariatric surgery, Adolescents, Patient Education Materials, Electronic education materials

## Abstract

**Objective:**

Adolescents who opt for metabolic and bariatric surgery (MBS) will use the internet to learn about the procedure. The objective of this study is to assess the suitability of electronic patient education materials (ePEM) of North American centers that perform adolescent bariatric surgery.

**Methods:**

Canadian and American bariatric centers that perform adolescent MBS were identified from the Metabolic and Bariatric Surgery Accreditation and Quality Improvement Program and Google web-based searches. Suitability of ePEM for the adolescent readership was evaluated using the Suitability Assessment of Materials (SAM).

**Results:**

Sixty-five centers were evaluated from June to July 2020 with 41% citing adolescent specific material. Six percent of the ePEM were evaluated as ‘not suitable’, 69% were evaluated as ‘adequate’, and 25% were evaluated as ‘superior’.

**Conclusion:**

Adequate ePEM scoring was obtained, but centers had little resources tailored to adolescent patients. Further research is needed to evaluate all the resources provided to adolescents (i.e., resources provided by the health team) to ensure the tools are appropriate for the adolescent readership.

**Innovation:**

This environmental scan provided insights to ePEM available for adolescents considering MBS.

## Highlights

• Electronic education materials are not tailored to meet the needs of adolescents.
• Inclusive and age-suitable materials are required to make informed decisions.
• Electronic education materials for bariatric surgery are available on most websites.
• Bariatric center websites need to consider the needs of adolescents.
• Few websites consider culture when discussing post-surgical diet recommendations.

## 1 Introduction

Obesity among adolescents is a public health concern worldwide [1]. In Canada, 12.9% of 10–14-year-olds and 18.2% of 15–17-year-olds are living with obesity [2]. In the United States, the prevalence of severe obesity (body mass index (BMI) ≥120% of the 95th percent for age and sex) among adolescents aged 12–15 and 16–19 years old is 7.5% and 9.5% retrospectively [3]. Adolescents with obesity are at an increased risk of associated complications including cardiovascular disease, type 2 diabetes, obstructive sleep apnea (OSA), polycystic ovary syndrome (PCOS), and degenerative joint disease that accompany obesity as a chronic disease [2,4,5]. Moreover, adolescents living with severe obesity have increased levels of stress, depressive symptoms, and reduced resilience [6]. An effective treatment for severe obesity in adolescents with related complications of the disease of obesity, is metabolic and bariatric surgery (MBS) [5]. Studies show improved complications associated with the disease of obesity and mental health following surgery [7,8]. The rate of current adolescents undergoing bariatric surgery in America has increased from 2.29 to 4.62 per 100,000 between 2010 and 2017 [9]. This is a small portion of the 4.5 million estimated adolescents living with obesity, and barriers limiting accessibility to surgery include race, socioeconomic status, and insurance coverage [10]. In contrast, Canada has reported 59 procedures being performed on adolescents aged 16 to 20 years old from 2009 to 2019 [11]. However, only one hospital offered the surgery during that time [11]. Limitations within Canada include long wait times for MBS [12], and the lack of empirical evidence of the rates of adolescents with severe obesity [13]. Other limitations that make it difficult for adolescents to choose MBS as a treatment option include a general lack of information and guidance by their medical providers, a lack of access to MBS related to insurance coverage and costs of the surgery, and an overall general stigma around MBS as a treatment for obesity [14].

Since the recognition of obesity as a disease by the American Medical Association in 2013, research on MBS has increased [15,16]. Many online resources are easily accessible to adult patients to help them understand and prepare for the psychological and physical changes after surgery. In contrast, MBS in adolescents remains a growing field of research [4,15]. Irrespective of weight, adolescents experience significant biological developments during puberty that result in heightened emotional states, changes in sleep patterns, social development, and the development of self-concept [17,18]. Furthermore, compared to their peers without obesity, children and adolescents living with obesity are more likely to have depression and depressive symptoms [5,19]. Adolescents who decide to undergo MBS have additional challenges that could reduce the adolescent patients' adherence [11,20,21]. These challenges include adjusting to postoperative routines such as specialized diet and exercise while navigating the customary challenges of puberty as they eventually transition to adulthood [11].

Many factors contribute to determining if an adolescent is a suitable candidate for MBS [5]. A multidisciplinary team of healthcare professionals typically work together with the adolescent and their caregiver in determining if they are eligible for the surgery [4,5,22]. Healthcare providers often provide patient education on MBS procedures and/or pre/post-surgical care guidance [23]. Patient education has been thought to improve adherence for adolescents with chronic diseases [24], and importantly, should describe the fundamental components specifically related to adolescent weight loss surgery [25]. Electronic patient education material (ePEM) are online resources that provide patients with information, advice and/or counselling on treatment, intended care, and aftercare [23]. Before considering MBS, patients are likely to investigate the process using online sources before visiting their physician [3,26,27]. Research has shown that adult bariatric patients will use the information regarding the surgical techniques and other patients' experiences on the websites affiliated to public hospitals to make their own decisions [27]. To date, few studies have evaluated online (electronic) patient materials for bariatric surgery [26,28,29]. A systematic review by Musbahi et al. [30] found that the average readability of adult materials was higher than the recommended patient reading level (grade 5–6) [30,31]. Overall, the quality of information on MBS online has been identified to be of poor quality [26]. Current evaluated ePEM focus on adults as the general audience, hence, it is clear that online educational resources for MBS that target adolescents require assessment to determine their appropriateness to adolescent bariatric patients and their families. Knowing that this age group spends substantial amounts of time using the internet to search for health information [18], it is important that the information related to MBS be tailored to adolescents. Therefore, the aims of this study were to conduct an environmental scan and qualitative assessment of existing ePEM on MBS catered for adolescents considering MBS. The main objective of this environmental scan was to identify and evaluate MBS resources (pre- and post-surgery) on websites for adolescents using a validated instrument Suitability Assessment Material (SAM) [32].

## 2 Method

### 2.1 PEM collection, inclusion, and classification

#### 2.1.1 Centers from an accredited list

A list of accredited MBS centers was found on the “Metabolic and Bariatric Surgery Accreditation and Quality Improvement Program” (MBSAQIP) [33] by searching for adolescent centers using the following search terms: “Adult and adolescent patients”, “Adolescent Center”, “Adolescent patients”, “Adolescent bariatric surgery” and “Teen bariatric surgery”. If no results were found, “Bariatric surgery” was entered as a keyword and the website was scanned for age-specific information.

#### 2.1.2 Centers not accredited

A Google web-based search was conducted from June to July 2020 to identify bariatric centers that are not accredited by the MBSAQIP but offer MBS for adolescents. Non-accredited centers that provided pediatric MBS were searched in the United States and Canada. Centers were in any of the 50 states of the United States of America, and those located within the 10 provinces and 3 territories of Canada were included; search terms included “Adolescent”, “Pediatric”, or “Teen”, and “Bariatric Surgery”.

The inclusion criteria for the centers were: 1) have a separate section (with a minimum of one paragraph) for adolescent MBS; 2) specifically indicate the target age group for adolescent MBS; 3) have a hospital/bariatric center located in the state/province/territory included in our search terms; and 4) written in English. Exclusion criteria included: 1) research article; 2) news-related articles; 3) links to physician (personal) websites; 4) webinars or workshops; 5) sites that were not accessible or found.

### 2.2 Electronic PEM

Electronic PEM (ePEM) for both adults and/or adolescents available on the centers' websites that provided information, advice or counseling about procedures and activities during the MBS process were included for analysis. In this paper, ePEM were classified as webpage text, informative videos, resource manuals, leaflets (i.e., PDF files) or slide decks (i.e., PowerPoint). Webpage texts were defined as a separate page dedicated specifically for an overview of MBS and may include links to other useful resources. An informative video was defined as a visual explanation of the types of bariatric surgeries available and patient stories. A resource manual was defined as electronic material that could be downloaded and/or printed and includes in-depth instructions on how to prepare for MBS and/or a thorough overview of post-surgical expectations. A leaflet was defined as a single-page handout (provided in a PDF format on the website) that provides patients and their families with a clear overview of what the process will involve leading up to/and on the day of the surgery and/or useful tips that adolescents should be aware of following their surgery. A slide deck was defined as additional material (more than one page, in .pptx or PDF format) providing information on pre- and post-surgery to prepare patients for MBS.

### 2.3 Content analysis

Each center's webpage was scanned for the following criteria available to adolescent patients and their families prior to surgery: indication of required “Family Support”, indication of “Multidisciplinary Team”, shared “Patient Success Story”, detailed “Surgical Procedures”.

### 2.4 SAM

SAM has been applied in previous studies to evaluate general healthcare-related materials [32,34,35] and used to assess pediatric and adolescent specific materials [[36], [37], [38], [39], [40]]. The tool consists of 22 questions that evaluate the suitability and readability of patient education materials. As SAM was designed prior to the diffuse of daily internet use to evaluate paper education materials, we adjusted SAM to evaluate electronic PEM designed specifically for adolescents on the 65 websites [32]. Each question was evaluated as “not suitable,” “adequate,” or “superior” based on specific SAM criteria for each category*: (1) Content, (2) Literacy Demand, (3) Graphic Illustrations, (4) Layout & Typography, (5) Learning Stimulation & Motivation, and (6) Cultural Appropriateness* [32]: (1) Content assesses if the purpose and scope of the material is clearly stated; (2) Literacy Demand assesses the reading level and the conversational style (active or passive voice) used; (3) Graphic Illustrations assesses the appropriateness of the cover graphic, and type of illustrations provided as visuals; (4) Layout & Typography assesses the layout of the material, font style and size; (5)Learning Stimulation & motivation assesses interaction in the text to keep the reader engaged; (6) Cultural Appropriateness assesses if the material language, images, and examples are appropriate for the cultural backgrounds of the target population [32]. Following SAM criteria, zero points were assigned when a material was evaluated as “not suitable,” one point when it was evaluated as “adequate,” and two points when it was evaluated as “superior” [32]. “Not applicable” or “N/A” was applied to a question when a criterion was not met [32]. The overall evaluation percentage score was calculated by dividing the total score of each material by the maximum total score (44) before multiplying it by 100%. As defined by SAM evaluation criteria, a material was considered “not suitable” when the percentage was between 0 and 39%, “adequate” when it was 40–69%, and “superior” when it was more than 70% [32].

### 2.5 Statistical analysis

Descriptive statistics were used to summarize the mean values (percentages) of both accredited and non-accredited MBS centers. Inferential statistics were used to determine the quality of the scores between the accredited and non-accredited bariatric centers. A two-tailed *t*-test (level of significance = 0.05) was performed using SAS Studio 3.8 on the SAM results to test for any significance in the difference of means in the suitability of the ePEM from accredited and non-accredited sites.

### 2.6 Interrater reliability

Two researchers (AH and YW) independently evaluated the same ePEM from the 20 sample sites using content criteria and SAM [32]. The researchers met to adjust for consistency in evaluating techniques [32] and discrepancies were reviewed and discussed by both raters to reach consensus. The mean of the two raters' results for scores was less than 5% of differences between total scores. The remaining bariatric centers (*n* = 45) were divided between two researchers (23 for AH and 22 for YW) and were evaluated individually for ePEM.

## 3 Results

### 3.1 Identifying sites

**Fig. 1:**
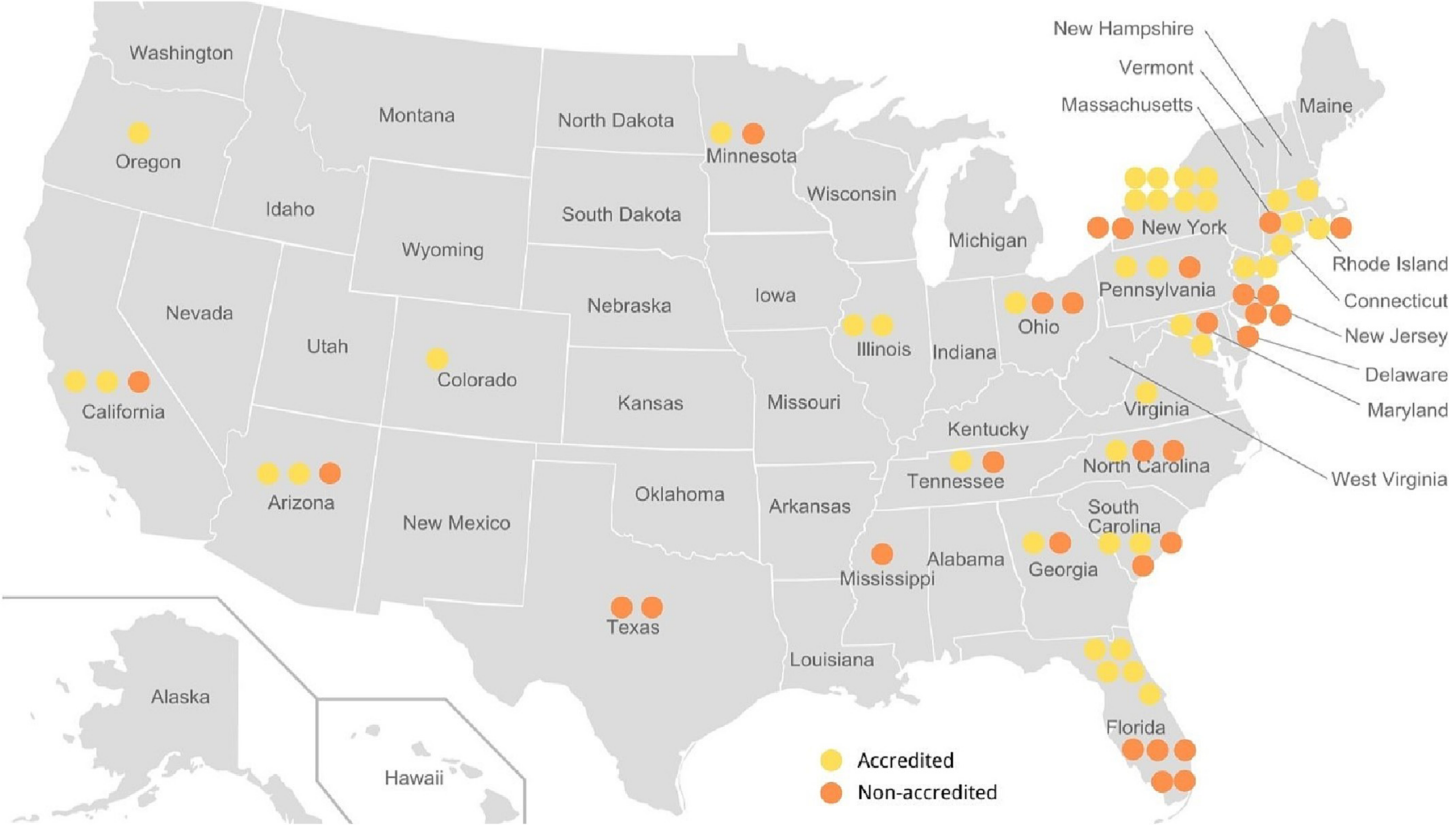
Bariatric Centers in the US providing adolescent bariatric surgeries by states. Yellow dots represent accredited centers. Orange dots represent non-accredited centers. (For interpretation of the references to colour in this figure legend, the reader is referred to the web version of this article.)

**Fig. 2:**
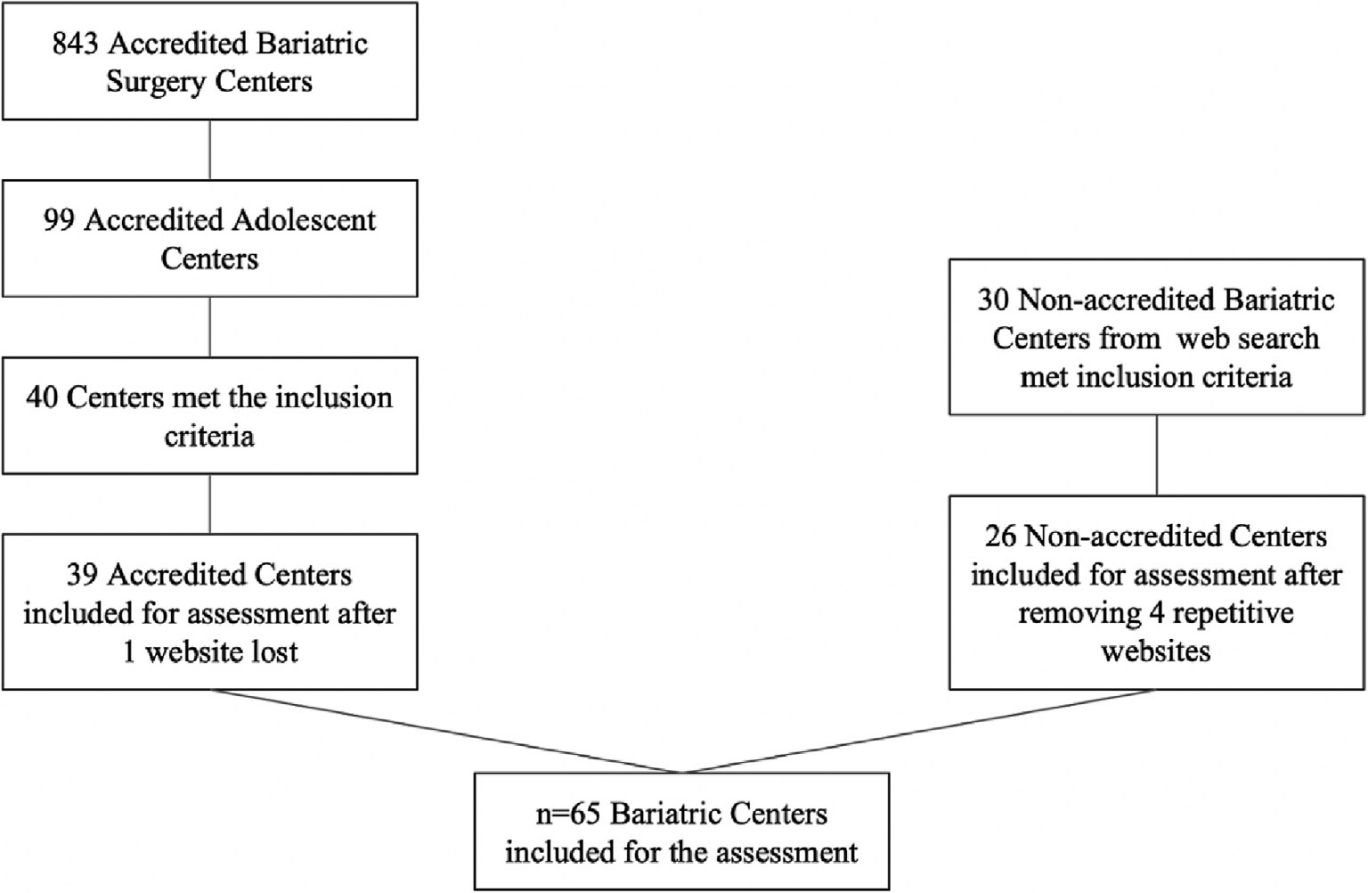
Flow chart of the selection process.

A web list of accredited bariatric centers was used and included a total of 843 sites for both adults and adolescents. This was narrowed down to 70 sites that contained ePEM for adolescents (Fig. 1): 40 accredited-centers sites and 30 non-accredited MBS centers that met all the eligibility criteria, however, four centers were omitted for content duplication on their websites (same company, but located in another state), and one center was omitted due to the website being inaccessible (website was undergoing changes). A final analysis was conducted on the websites of the remaining 65 centers (Fig. 2), which included 39 accredited and 26 non-accredited sites.

### 3.2 Content analysis

Table 1 provides an overview of the content analysis for accredited and non-accredited sites. Majority of the sites stated a multidisciplinary team was involved in the care of the patients (94.9% accredited sites, 80.0% non-accredited sites). Majority (94.9%, 84.0%) of all the accredited and non-accredited sites, respectively, provided information on metabolic and bariatric surgical procedures, with only 28.2% of the accredited centers and 61.5% of the non-accredited site have materials targeting specifically to adolescent group. Finally, 89.7% of the accredited sites had ePEM for pre-surgery, while only 53.8% of non-accredited sites provided ePEM on pre-surgery. However, only 67.6% and 43.5% of accredited and non-accredited sites respectively, had post-surgery materials.

**Table 1:** The percentage of accredited and non-accredited bariatric centres that included each criterion.

Adolescent Bariatric Centers	Accredited		Non-accredited	
Criteria	Yes	No	Yes	No
Family Support	71.8%	28.2%	65.4%	34.6%
Multidisciplinary HCP Team	94.9%	5.1%	80.0%	20.0%
Patient Success Stories (Text)	18.0%	82.1%	61.5%	38.5%
Patient Success Stories (videos)	20.5%	79.5%	69.2%	30.8%
Surgical Procedures (general)	94.9%	5.1%	84.0%	16.0%
Surgical Procedures (adult side)	76.9%	23.1%	64.0%	36.0%
Surgical Procedures (teens)	28.2%	71.8%	61.5%	38.5%
Pre-surgery Education Material	89.7%	10.3%	53.8%	46.2%
Post-surgery Education Material	67.6%	32.4%	43.5%	56.5%

### 3.3 SAM

**Fig. 3:**
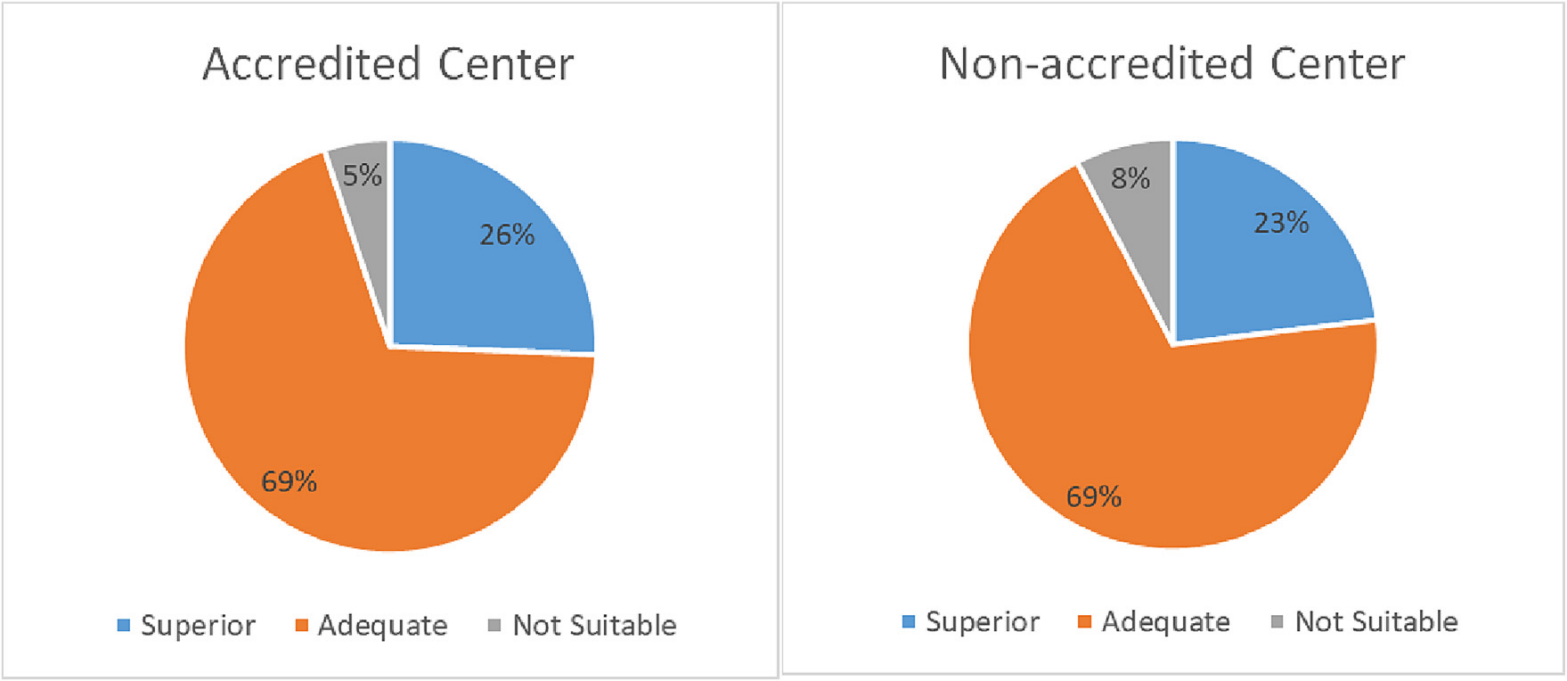
a. The distribution of superior, adequate, and not suitable ePEM among accredited centers (*n* = 39); b. The distribution of superior, adequate, and not suitable ePEM among non-accredited centers (*n* = 26).

The total score calculated from all the 39 accredited sites (60.7%) was not significantly different from the total score derived from the 26 non-accredited sites (58.0%) (*p* = 0.46), with 5.1% (*n* = 2) of the ePEM scored as ‘not suitable’, 69.2% (*n* = 27) scored as ‘adequate’, and 25.6% (*n* = 10) scored as ‘superior’ for accredited sites (Fig. 3a). Total scores derived from the 26 non-accredited sites found 8% (n = 2) of ePEM “not suitable”, 69% (*n* = 18) as “adequate”, and 23% (*n* = 6) as “superior” (Fig. 3b).

The highest rating for both accredited and non-accredited ePEM was (4) *Layout and Typography* category, which was found superior with a score of 5.4 (90.3%) (score of 5.5 (91.9%) for all accredited sites and 5.3 (87.8%) for all non-accredited sites). The lowest score was the (3) *Graphic Illustrations* (lists, tables, charts) category, 4.5 (45.7%) (average score of 4.4 (44.6%) for accredited sites and 4.8 (48.8%) for non-accredited sites), which was rated adequate. Many sites received N/A for (3) *Graphic Illustration*, as the ePEM did not qualify for the criteria specific for this category (Table 2).

**Table 2:** The average score of each category from SAM for accredited and non-accredited centers.

	Accredited		Non-accredited	
SAM Criteria	Score	Percentage	Score	Percentage
Content	5.1	64.1%	4.5	56.7%
Literacy Demand	6.1	61.3%	5.5	54.6%
Graphic Illustrations	4.5	44.6%	4.9	48.8%
Layout & Typography	5.5	91.9%	5.3	87.8%
Learning Stimulation & Motivation	3.3	54.7%	3.2	53.2%
Cultural Appropriateness	2.2	54.5%	2.2	54.8%
Overall Score (Out of 44)	26.7	60.7%	25.5	58.0%

## 4 Discussion and conclusion

### 4.1 Discussion

This study found that the majority of bariatric centers have a “multidisciplinary team” involved in guiding adolescent patients and their families throughout the surgical procedure. Most bariatric centers (71.8% accredited, 65.4% unaccredited) included “Family Support” as a requirement for admission into the surgical program. The materials available on MBS center websites provide sufficient information on general procedures for both accredited (94.9%) and non-accredited sites (84.0%). However, the information concerning metabolic and bariatric surgical procedure revealed that less than half of the accredited bariatric centers (28.2%) include resources customized for adolescents, whereas non-accredited centers provide twice as many resources (61.5%) for adolescents. This study brings to light the quality of resources available to adolescent metabolic and bariatric surgery candidates on the MBS centers' websites and emphasizes that ePEM should be developed to help adolescent metabolic and bariatric patients and their parents to address their unique challenges and improve their comprehension and adherence [11,20,21].

Applying the SAM tool [32] to further evaluate the suitability of the online materials found that out of the 39 accredited sites, 5.1% (*n* = 2) were “not suitable”, 69.2% (*n* = 27) were “adequate”, and 25.6% (*n* = 10) were “superior”; with similar findings for the 26 non-accredited sites. While an overall “adequate” rating was achieved for all MBS sites (accredited and non-accredited), this study supports the need for age-appropriate ePEM that are both appealing to adolescents and easy to understand, that will help adolescent patients and their families understand and better prepare for the emotional and physical complexity of undergoing MBS. Using SAM criteria to determine suitability [32], websites that were considered suitable for adolescents contained appropriate images, or videos of adolescents in larger bodies within a medical setting, had a webpage that was friendly and inviting, used plain terminology to describe procedures and had appropriate resources that were within a grade 5 reading level. Websites that were considered not suitable contained images of adults within a medical setting, was text heavy with an overuse of medical jargon to describe procedures, had no images to help describe the surgical procedures, and was written at a grade 9 reading level or above. Other studies that used the SAM tool [32] to evaluate patient materials for adults with chronic diseases, whether it be for prostate cancer [35], age-related hearing [41], colorectal cancer (assessed with a modified SAM checklist) [23], or cancer education [34,42], found similar results where an overall “adequate” scoring was found. Studies that utilized SAM caution that the overall “adequate” scoring obtained is due to the degree of ‘suitability’ found within the six SAM categories and noted that materials with a “not suitable” scoring for *Literacy Demand* will not be effective, as the target audience will not comprehend the materials [34,35,43]. Only one study conducted by Monton et al. [44] using the SAM tool [32] to evaluate patient online materials for adults, found the materials “not suitable” [44]. To date, only the study by Sadeghi et al. [36] utilized the SAM tool [32] for assessing PEM for an adolescent audience [36]. Interestingly this study found that materials had a “superior” score when targeting a general audience, which was later increased after the materials were tailored to the target the adolescent audience [36].

When investigating the six categories of SAM we found that each category had a variation of average scores that are comparable to other studies [32,34,35,43]. An overall total score of 58.6% for *Literacy Demand* was achieved for all ePEM, indicating that the materials are adequate and suggest that the information content is suitable for a grade 6 to grade 8 reading comprehension [32].

This environmental scan found that many sites were not able to be rated for *Graphic Illustrations* and were rated N/A. While graphics are common for printed PEM to attract patient interest, we found the webpages to be text heavy with minimal graphics, tables, and charts making it difficult to translate these criteria into our evaluation as most graphics, tables, and charts were found on additional resource pages. This finding is comparable to other studies using SAM [32,34,43]. Despite the “adequate” score achieved, the suitability of the illustrations varied within materials. Conversely, *Layout and Typography* presented the strongest evaluation out of the six SAM criteria [32]. This evaluation was the easiest to achieve for majority of the bariatric center's online resources due to the simplicity of its requirements, which were based on font type, font size and subheadings. The rating is comparable to previous studies using SAM [32] to evaluate patient education materials [35,43].

*Cultural Appropriateness* is considered pertinent when it provides images and examples that represent the socioeconomic and ethnic backgrounds of the intended audience [32]. It is an important aspect to consider when designing ePEM as cultural health beliefs and practices differ among ethnic backgrounds [35]. When evaluating the cultural appropriateness of the ePEM, this study considered the demographic representation of that state, province, or territory. Studies on printed education materials show that positive behaviour changes increased when the message was delivered in a culturally relevant manner depicting images of the targeted ethnic group [35]. This environmental scan found an average score of 59.6%, which was interpreted as a neutral presentation of cultural images and foods on each site. Many sites had images of people from various ethnic backgrounds and provided ePEM in more than one language, but there was no significant indication of cultural foods or menus to provide a sense consideration of the diversity among MBS patients. The neutrality of cultural depictions reflects the current racial group that undergo MBS, which are White adolescents (45.0%) [9]. Despite the evidence that MBS is underutilized by minority groups [9], more emphasis on Black and Hispanic representation in ePEM from American MBS centers should be considered, as current research has shown high rates of obesity in Hispanic (25.6%) and Black (24.2%) children aged 2 to 19 years old [9]. Providing some information (i.e., menus) that demonstrate examples of post-surgery meals that are aligned with diverse cultural foods should be taken into consideration as analyzed National Health and Nutrition Examination Survey (NHANES) 2011–2012 data has shown that the age-adjusted prevalence of obesity among adults of most racial groups excluding Asian Americans is over 30% in the United States [45].

Overall, the SAM results revealed the MBS centers provide adequate, suitable, and readable educational material on their websites. However, this does not imply that the websites evaluated provide adequate information to readers. This adequacy is due to the imbalance of “not suitable” and “superior” content presented in each site. Many of the websites lack certain SAM criteria while meeting the expectations for another [32]. Therefore, the degree of suitability and readability in each website vary greatly. This has been observed similarly in other studies that evaluated PEM [34,35,43], where authors cautioned interpretation of the overall “adequate” scoring due to the degree of ‘suitability’ found within the six SAM categories.

### 4.2 Innovation

Although the SAM tool was designed for printed material and illustrations, it has been used for video and audiotaped instructions [32]. Thus, the malleability of the SAM tool allows for adaptability to the current era of electronic education materials. With the increase in adolescents with severe obesity in North America [10], and the increase of MBS as a treatment option [10], this environmental scan captured the first exposure of ePEM offered for adolescent bariatric surgery.

This environmental scan was comprehensive as it included both the accredited sites from MBSAQIP and the non-accredited sites from a direct web search. However, it is important to highlight that the environmental scan was conducted on bariatric centers located in North America, primarily the United States, as there are limited bariatric programs that are catered for adolescents within Canada. Furthermore, the assessment tool, SAM [32], that was used in this study was developed for printed materials. While only a few studies have used SAM to evaluate adolescent-focused educational tool [36,37], this study contributes to the use of the tool in adolescent-targeted PEM and demonstrates the need to have adolescent appropriate suitability tools. Some of the criteria in the tool are not suitable for electronic material, and therefore influence the results obtained. Since rating content is dependent on a certain number of criteria being met for each question, having criteria that were not compatible with the electronic format of PEM may have impacted the results.

A limitation of this study is the insufficient access to all resources provided by the bariatric surgery centers for adolescent surgeries. Centers may offer additional printed resources to patients that have had in-person consultations, which are not available to the public. These materials may be of higher quality and more suitable for the target audience. Moreover, it is possible adolescents are referred to bariatric centers primarily for adults and therefore these sites would not be included in this study given our inclusion criteria.

### 4.3 Conclusion

This environmental scan revealed that MBS centers have adequate electronic resources available for adult patients on their websites, but the electronic patient education materials available for adolescent patients were not age-appropriate for the adolescent population. It is worth considering that the MBS centers offering adolescent care provide more suitable materials during in person consultations. A future investigation that entails contacting MBS centers to obtain access to all ePEM provided to adolescent patients is required to gain a comprehensive analysis of the suitability of all materials for adolescent bariatric patients. This study contributes to the growing literature on MBS for adolescents.

## Funding Information

This work was supported by the 10.13039/501100005247University of British Columbia through TC start-up funds.

## CRediT authorship contribution statement

**Yolanda N. Wang:** Methodology, Investigation, Formal analysis, Writing – original draft. **Alexandra J. Heidl:** Investigation, Writing – original draft. **Patricia M. Angeles:** Formal analysis, Writing – original draft. **Biagina-Carla Farnesi:** Writing – review & editing. **Angela S. Alberga:** Conceptualization, Supervision, Validation, Writing – review & editing. **Tamara R. Cohen:** Conceptualization, Supervision, Validation, Writing – review & editing.

## Declaration of Competing Interest

The authors declare that they have no known competing financial interests or personal relationships that could have appeared to influence the work reported in this paper.
